# Summer Diseases

**Published:** 1893-09

**Authors:** 


### SUMMER DISEASES.

THE hot weather is here, and with it the beginning of bowel troubles. While the heat is a factor in the causation of some of these troubles, because of its depressing effect when long continued, yet it is only a small factor. The principal cause is the kind of food ingested, but more particularly the quantity, quality, and character of the food. An excessive amount taken, or when taken green, or overripe, or stale, will act as an irritant to the mucous membrane of the alimentary tract, or poison the blood, and thus bring on gastric or enteric troubles.

In the treatment of these cases the physician must impress emphatically upon the patient's mind, and that of the attendant, that diet and hygiene are the essential factors in the cure and in preventing the occurrence of another attack. At the time a simple diet must be taken. Easily digestible fluids and solids, known to agree with the patient, such as milk, eggs, toast, etc., should be given, and cool or hot water, as the appetite may crave—at least, the temperature should be such as not to cause pain in the stomach or bowels.

On general principles, the bowels should be emptied by a cathartic which will not be drastic in its nature, such as salts of magnesia, with a little camphorated tincture of opium, or a few drops of the extract of hyoscyamus, after which the hyoscyamus and a little tincture of opium, sufficient to control the pain and irritation, should be given, and entire rest should be enjoined. Flushing the colon, in some cases, is essential, and if there is periodicity, a sufficient quantity of quinine should be given to overcome the malaria. The skin should be kept clean and protected from the sudden changes from draughts of air. Thin flannel should be worn next to the skin. With attention to the food, skin, and hygiene, but little medication will be required, and the mortality lessened.—*Kansas Medical Journal.*

---

BERLIN has 183 polyclinics. Lately a number of medical men met to discuss the situation, which was regarded as militating against the profession. A committee was appointed to study the question and report as to the proper steps to check the encroachments of the polyclinics.

